# Trends in Characteristics, Mortality, and Other Outcomes of Patients With Newly Diagnosed Cirrhosis

**DOI:** 10.1001/jamanetworkopen.2019.6412

**Published:** 2019-06-28

**Authors:** Eric S. Orman, Anna Roberts, Marwan Ghabril, Lauren Nephew, Archita Desai, Kavish Patidar, Naga Chalasani

**Affiliations:** 1 Division of Gastroenterology and Hepatology, Indiana University School of Medicine, Indianapolis; 2 Regenstrief Institute, Inc, Indianapolis, Indiana

## Abstract

**Importance:**

Changes in the characteristics of patients with cirrhosis are likely to affect future outcomes and are important to understand in planning for the care of this population.

**Objective:**

To identify changes in demographic and clinical characteristics and outcomes in patients with newly diagnosed cirrhosis.

**Design, Setting, and Participants:**

A retrospective cohort study of patients with a new diagnosis of cirrhosis was conducted using the Indiana Network for Patient Care, a large statewide regional health information exchange, between 2004 and 2014. Patients with at least 1 year of continuous follow-up before the cirrhosis diagnosis were followed up through August 1, 2015. The analysis was conducted from December 2018 to January 2019.

**Exposures:**

Age, cause of cirrhosis, and year of diagnosis.

**Main Outcomes and Measures:**

Overall rates for mortality, liver transplant, hepatocellular carcinoma, and hepatic decompensation (composite of ascites, hepatic encephalopathy, or variceal bleeding).

**Results:**

A total of 9261 patients with newly diagnosed cirrhosis were identified (mean [SD] age, 57.9 [12.6] years; 5109 [55.2%] male). A 69% increase in new diagnoses occurred over the course of the study period (620 in 2004 vs 1045 in 2014). The proportion of those younger than 40 years increased by 0.20% per year (95% CI, 0.04% to 0.36%; *P* for trend = .02), and the proportion of those aged 65 years and older increased by 0.81% per year (95% CI, 0.51% to 1.11%; *P* for trend < .001). The proportion of patients with alcoholic cirrhosis increased by 0.80% per year (95% CI, 0.49% to 1.12%), and the proportion with nonalcoholic steatohepatitis increased by 0.59% per year (95% CI, 0.30% to 0.87%), whereas the proportion with viral hepatitis decreased by 1.36% per year (95% CI, −1.68% to −1.03%) (*P* < .001 for all). In patients younger than 40 years, 40 to 64 years, and 65 years and older, mortality rates were 6.4 (95% CI, 5.4 to 7.6), 9.9 (95% CI, 9.5 to 10.4), and 16.2 (95% CI, 15.2 to 17.2) per 100 person-years, respectively (*P* < .001). Mortality rates decreased during the study period (11.9 [95% CI, 10.7-13.1] per 100 person-years in 2004 vs 10.0 [95% CI, 8.1-12.2] per 100 person-years in 2014; annual adjusted hazard ratio, 0.87 [95% CI, 0.86 to 0.88]) and were lower in those with alcoholic cirrhosis compared with patients with viral hepatitis (adjusted hazard ratio, 0.89 [95% CI, 0.80 to 0.98]). Rates of hepatocellular carcinoma were low in patients younger than 40 years (0.5 [95% CI, 0.2 to 0.9] per 100 person-years). Liver transplant rates were low throughout the study period (0.3 [95% CI, 0.3-0.4] per 100 person-years). In patients with compensated cirrhosis, rates of hepatic decompensation were lower in patients younger than 40 years (adjusted subhazard ratio 0.78; 95% CI, 0.62 to 0.99) and in patients with nonalcoholic steatohepatitis (adjusted subhazard ratio, 0.51; 95% CI, 0.43 to 0.60).

**Conclusions and Relevance:**

The population of patients with newly diagnosed cirrhosis in Indiana has experienced changes in the age distribution and cause of cirrhosis, with decreasing mortality rates. These findings support investment in the prevention and treatment of alcoholic liver disease and nonalcoholic steatohepatitis, particularly in younger and older patients. Additional study is needed to identify the reasons for decreasing mortality rates.

## Key Points

**Question:**

What are contemporary trends in the characteristics and outcomes of patients with newly diagnosed cirrhosis?

**Findings:**

In this cohort study of 9261 patients with newly diagnosed cirrhosis from 2004 to 2014, the proportions of patients younger than 40 years and those 65 years and older increased significantly, as did the proportions with alcoholic cirrhosis and nonalcoholic steatohepatitis. Mortality decreased over time.

**Meaning:**

The population of patients with newly diagnosed cirrhosis is facing changes in demographic characteristics, clinical features, and outcomes that may affect future care.

## Teaser

This cohort study examines changes in the demographic and clinical characteristics and outcomes of patients with newly diagnosed cirrhosis.

## Introduction

Cirrhosis is the end point of a variety of chronic liver diseases and can lead to such complications as ascites, variceal bleeding, and hepatic encephalopathy. These complications contribute to cirrhosis being the 12th leading cause of death in the United States.^1^ Cirrhosis is also the primary risk factor for hepatocellular carcinoma (HCC), which is increasing in incidence and associated mortality.^2,3^

These outcomes are influenced by patient demographic and clinical characteristics. For example, increases in cirrhosis-related mortality in young adults have been ascribed to alcohol use.^4^ In contrast, cirrhosis due to nonalcoholic steatohepatitis (NASH) is more commonly diagnosed in older patients, who have lower rates of transplant and greater mortality while waiting for a transplant.^5^ Viral hepatitis, which in the United States is most prevalent among baby boomers, is strongly associated with HCC.^6,7^ As these characteristics change on a population level, we would expect to see corresponding changes in outcomes. Recent data show increases in alcohol use disorders in the general population as well as an increase in alcoholic cirrhosis–related mortality.^4,8^ The epidemics of obesity and diabetes have resulted in increasing frequency of NASH, with corresponding increases in transplant referrals.^9,10^ With respect to viral hepatitis, the expanding availability of effective antiviral agents may lead to improved outcomes.^11^ In contrast to these recent changes in the United States, much of our knowledge of the natural history of cirrhosis is derived from older studies and studies performed elsewhere.^12,13,14^

Understanding changes in the demographic characteristics, clinical characteristics, and outcomes of patients with cirrhosis is therefore critical to allocating resources and prioritizing future research. An improved understanding of the contemporary natural history of cirrhosis can also better inform medical decision making. To explore these trends, we used data from the Indiana Network for Patient Care, a statewide repository of clinical and administrative data from a variety of sources. We hypothesized that we would be able to identify trends in patient characteristics with implications for outcomes.

## Methods

### Study Design

This study was approved by the Indiana University institutional review board. Informed consent was waived because this study used deidentified retrospective data. This report follows the Strengthening the Reporting of Observational Studies in Epidemiology (STROBE) reporting guideline.^15^ We performed a retrospective cohort study of patients with newly diagnosed cirrhosis between 2004 and 2014 in the Indiana Network for Patient Care, the largest interorganizational clinical data repository in the United States, connecting more than 46 000 clinicians, 16 000 practices, and 110 hospitals across Indiana. It contains inpatient and outpatient data from more than 14 million patients, including both administrative and clinical data, as well as death certificate information for patients who die inside and outside Indiana.^16^ Claims-based definitions based on *International Classification of Diseases, Ninth Revision, Clinical Modification* or *Current Procedural Terminology* codes are provided in eTable 1 in the Supplement. Patients were included if they (1) were aged 18 years or older at the time of diagnosis and (2) had at least 2 separate claims for cirrhosis, according to previously validated codes.^17^ To ensure that these were incident cirrhosis diagnoses (ie, new diagnoses), we required at least 1 year of continuous follow-up in the Indiana Network for Patient Care before the first cirrhosis claim. We excluded patients with a claim for any solid-organ transplant during this 1-year period.

Patients were followed up in the cohort from the time of the initial cirrhosis claim until the first of the following events: (1) death, (2) liver transplant, or (3) administrative censoring on August 1, 2015. This date was chosen to avoid irregularities due to the transition to *International Classification of Diseases, Tenth Revision*. The 1-year run-in period was not counted as person-time in the cohort. The analysis was conducted from December 2018 to January 2019.

### Variables

The primary variables of interest were age at the time of diagnosis and cause of cirrhosis. Outcomes included mortality, liver transplant, HCC, and hepatic decompensation. Incidence estimates of HCC and decompensation excluded patients with these complications in the first 180 days of follow-up, because these are likely to be prevalent complications.

The cause of cirrhosis was defined on the basis of the presence of diagnostic codes and laboratory results during either the run-in period or follow-up time (eTable 1 in the Supplement). For those with viral hepatitis and another cause (eg, alcohol), the cause was considered to be viral hepatitis. Patients with alcohol and another nonviral cause were considered to have alcohol-induced cirrhosis. Where available, validated coding algorithms were used.^18,19^ Patients without 1 of the listed diagnoses were considered to have NASH or another cause of liver disease.

Comorbidities were assessed only during the run-in period and were determined according to the Deyo modification of the Charlson comorbidity index.^20^ The liver disease categories were excluded to avoid double counting the cirrhosis complications.^21^ Ascites during follow-up was defined by the presence of a code for ascites or paracentesis or by the presence of ascites laboratory specimen. The presence of hepatic encephalopathy, variceal bleeding, and HCC during follow-up was determined on the basis of diagnostic codes. Decompensation was defined by the occurrence of ascites, hepatic encephalopathy, or variceal bleeding during follow-up. These complications of liver disease were considered to be prevalent (ie, present at cirrhosis diagnosis) when they were identified in the first 180 days of follow-up.

### Statistical Analysis

Categorical variables were described using counts and percentages, and bivariable comparisons were performed with Pearson χ^2^ test. Continuous variables were described using means and standard deviations, and comparisons were made using 1-way analysis of variance. Temporal linear trends in proportions were assessed using the Cochran-Armitage test for trend. We used survival analysis to examine differences in outcomes based on age and cause of cirrhosis and to examine temporal trends in outcomes. Mortality outcomes were compared using the log-rank test and Cox proportional hazards regression, and other outcomes were compared using competing risk regression, accounting for the competing risk of death that would prevent the outcome of interest (eg, death prevents a patient from undergoing liver transplant).^22^ Multivariable models included age, sex, cause of cirrhosis, decompensation at baseline, Charlson comorbidity index, health insurance, and year of cirrhosis diagnosis. Two-sided tests were used, and *P* < .05 was considered statistically significant. Analyses were performed using Stata statistical software version 13 (StataCorp).

### Sensitivity Analyses

We repeated analyses of trends and outcomes for causes of cirrhosis after restricting the NASH definition. This definition of NASH was based on the presence of a NASH diagnostic code or the presence of metabolic syndrome without an alternative cause of cirrhosis. Metabolic syndrome was defined according to guidelines established by the National Cholesterol Education Program Adult Treatment Panel III,^23^ according to the presence of at least 3 of the following factors: obesity, dyslipidemia, hypertension, and impaired fasting glucose or diabetes, with corresponding diagnostic codes.^24^

## Results

### Patient Characteristics

The cohort included 9261 patients with cirrhosis, of whom 585 (6.3%) were younger than 40 years, 6027 (65.1%) were aged 40 to 64 years, and 2649 (28.6%) were aged 65 years and older. The mean (SD) age was 57.9 (12.6) years; 5109 patients (55.2%) were men. The median (interquartile range) duration of follow-up was 2.1 (0.7-4.5) years. Overall patient characteristics and comparisons between the age groups are shown in Table 1. The younger and older groups had higher proportions of women compared with those aged 40 to 64 years (285 [48.7%] and 1366 [51.6%] vs 2501 [41.5%]). The leading causes of cirrhosis were alcohol in the group younger than 40 years, viral hepatitis in the group aged 40 to 64 years, and NASH in the group aged 65 years and older. At baseline, 3149 patients (34.0%) had decompensation, and of those, 2111 (67.0%) had ascites and 1613 (51.2%) had hepatic encephalopathy (similar across age groups). Hepatocellular carcinoma was present in 439 patients (4.7%) and was more common among older patients (174 [6.6%] of those aged ≥65 years vs 6 [1.0%] of those aged <40 years). Older patients also had a greater burden of comorbidities; 1425 (53.8%) of those aged 65 years and older had at least 2 comorbidities (excluding their liver disease) compared with 2156 (35.8%) of those aged 40 to 64 years and 148 (25.3%) of those younger than 40 years. The top payers were Medicaid for those younger than 40 years (199 patients [36.7%]), commercial insurance for those aged 40 to 64 years (1664 patients [31.0%]), and Medicare for those aged 65 years and older (2226 patients [89.8%]).

**Table 1.** Baseline Characteristics

Characteristic	Patients, No. (%)				*P* Value
	All Ages	<40 y	40-64 y	≥65 y	
Total	9261	585 (6.3)	6027 (65.1)	2649 (28.6)	
Male	5109 (55.2)	300 (51.3)	3526 (58.5)	1283 (48.4)	<.001
Cause of cirrhosis					
Alcohol	3103 (33.5)	270 (46.2)	1976 (32.8)	857 (32.4)	<.001
Viral	3610 (39.0)	167 (28.5)	2995 (49.7)	448 (16.9)	
Nonalcoholic steatohepatitis or other	2308 (24.9)	128 (21.9)	940 (15.6)	1240 (46.8)	
Autoimmune or cholestatic	240 (2.6)	20 (3.4)	116 (1.9)	104 (3.9)	
Decompensated	3149 (34.0)	219 (37.4)	2018 (33.5)	912 (34.4)	.13
Ascites^a^	2111 (67.0)	147 (67.1)	1362 (67.5)	602 (66.0)	.73
Hepatic encephalopathy^a^	1613 (51.2)	117 (53.4)	1039 (51.5)	457 (50.1)	.63
Hepatocellular carcinoma	439 (4.7)	6 (1.0)	259 (4.3)	174 (6.6)	<.001
Charlson comorbidity index					
0	3711 (40.1)	322 (55.0)	2656 (44.1)	733 (27.7)	<.001
1	1821 (19.7)	115 (19.7)	1215 (20.2)	491 (18.5)	
2-3	1939 (20.9)	81 (13.8)	1152 (19.1)	706 (26.7)	
≥4	1790 (19.3)	67 (11.5)	1004 (16.7)	719 (27.1)	
Insurance					
Commercial	1954 (23.3)	149 (27.5)	1664 (31.0)	141 (5.7)	<.001
Medicare	3566 (42.6)	71 (13.1)	1269 (23.7)	2226 (89.8)	
Medicaid	1745 (20.8)	199 (36.7)	1488 (27.8)	58 (2.3)	
Other	1115 (13.3)	123 (22.7)	939 (17.5)	53 (2.1)	

^a^ Percentage of patients with decompensated cirrhosis.

Comparisons of the different causes of cirrhosis are shown in Table 2. The group with viral hepatitis had the highest proportion of men (2237 [62.0%]), and the group with autoimmune or cholestatic disease had the highest proportion of women (141 [58.7%]). Patients with alcoholic cirrhosis and NASH had higher proportions of baseline decompensation (1247 [40.2%] and 893 [38.7%], respectively) than did patients with viral hepatitis (945 [26.2%]). At baseline, HCC was present in 265 (7.3%) of those with viral hepatitis, compared with only 70 (2.3%) of those with alcoholic cirrhosis and 92 (4.0%) of those with NASH. The comorbidity burden was greatest among those with NASH.

**Table 2.** Baseline Characteristics According to Cause of Cirrhosis

Characteristic	Patients, No. (%)				*P* Value
	Alcohol (n = 3103)	Viral (n = 3610)	NASH or Other (n = 2308)	Autoimmune or Cholestatic (n = 240)	
Age, mean (SD), y	56.7 (12.4)	54.6 (9.3)	64.5 (14.4)	60.8 (13.9)	<.001
Male	1666 (53.7)	2237 (62.0)	1107 (48.0)	99 (41.3)	<.001
Decompensated	1247 (40.2)	945 (26.2)	893 (38.7)	64 (26.7)	<.001
Ascites^a^	839 (67.3)	723 (76.5)	508 (56.9)	41 (64.1)	<.001
Hepatic encephalopathy^a^	684 (54.9)	383 (4.5)	514 (57.6)	32 (50.0)	<.001
Hepatocellular carcinoma	70 (2.3)	265 (7.3)	92 (4.0)	12 (5.0)	<.001
Charlson comorbidity index					
0	1325 (42.7)	1672 (46.3)	598 (25.9)	116 (48.3)	<.001
1	671 (21.6)	705 (19.5)	407 (17.6)	38 (15.8)	
2-3	636 (20.5)	705 (19.5)	554 (24.0)	44 (18.3)	
≥4	471 (15.2)	528 (14.6)	749 (32.5)	42 (17.5)	
Insurance					
Commercial	722 (26.4)	757 (22.8)	407 (19.2)	68 (33.0)	<.001
Medicare	1118 (40.9)	1011 (3.4)	1330 (62.9)	107 (51.9)	
Medicaid	489 (17.9)	1044 (31.4)	202 (9.6)	10 (4.9)	
Other	403 (14.8)	515 (15.5)	176 (8.3)	21 (10.2)	

Abbreviation: NASH, nonalcoholic steatohepatitis.

^a^ Percentage of patients with decompensated cirrhosis.

### Trends in Baseline Characteristics

Between 2004 and 2014, new diagnoses of cirrhosis increased by 69% (620 in 2004 vs 1045 in 2014). The proportion of those younger than 40 years increased by 0.20% per year (95% CI, 0.04% to 0.36%; *P* for trend = .02), whereas the proportion of those aged 65 years and older increased by 0.81% per year (95% CI, 0.51% to 1.11%; *P* for trend < .001) (Figure, A). The mean (SD) age increased from 56.0 (13.1) years in 2004 to 59.1 (13.1) years in 2014. Annual changes in baseline characteristics are shown in Table 3. The sex distribution did not change over time, but the cause of liver disease did change. The proportion of liver cirrhosis due to alcohol increased by 0.80% (95% CI, 0.49% to 1.12%) and the proportion due to NASH increased by 0.59% per year (95% CI, 0.30% to 0.87%), whereas the proportion due to viral hepatitis decreased by 1.36% per year (95% CI, −1.68% to −1.03%) (*P* < .001 for all) (Figure, B). The increase in alcohol-related cirrhosis was seen in all age groups and was most pronounced in patients younger than 40 years (2.32% per year; 95% CI, 1.02% to 3.62%) (Table 3). There was an increase in baseline decompensation by 1.80% per year (95% CI, 1.49% to 2.12%) across age groups, with greater increases for ascites (1.84% per year; 95% CI, 1.56% to 2.12%) compared with hepatic encephalopathy (0.85% per year; 95% CI, 0.59% to 1.10%). Comorbidity burden also increased over time, particularly in those aged 40 to 64 years and in those aged 65 years and older, with the proportion of patients with 4 or more comorbidities increasing by 0.39% per year (95% CI, 0.08% to 0.70%) and 0.75% per year (95% CI, 0.20% to 1.30%), respectively. The prevalence of HCC did not change over time.

**Figure.**
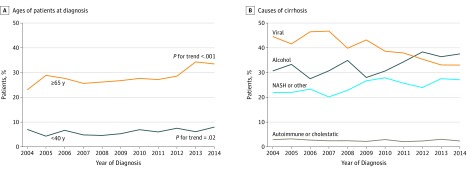
Temporal Trends in Ages of Patients at Diagnosis and in Causes of Cirrhosis Temporal trends in ages of patients at diagnosis (A) and in causes of cirrhosis (B). NASH indicates nonalcoholic steatohepatitis.

**Table 3.** Temporal Trends in Patient Characteristics

Characteristic	All Ages		<40 y		40-64 y		≥65 y	
	Change per Year, % (95% CI)	*P* Value	Change per Year, % (95% CI)	*P* Value	Change per Year, % (95% CI)	*P* Value	Change per Year, % (95% CI)	*P* Value
Male	−0.11 (−0.44 to 0.23)	.53	0.06 (−1.24 to 1.36)	.92	−0.08 (−0.49 to 0.32)	.69	0.14 (−0.48 to 0.76)	.66
Cause of cirrhosis								
Alcohol	0.80 (0.49 to 1.12)	<.001	2.32 (1.02 to 3.62)	<.001	0.70 (0.31 to 1.09)	<.001	0.63 (0.04 to 1.21)	.03
Viral	−1.36 (−1.68 to −1.03)	<.001	−2.88 (−4.06 to −1.70)	<.001	−1.30 (−1.72 to −0.89)	<.001	−0.07 (−0.53 to 0.39)	.77
Nonalcoholic steatohepatitis or other	0.59 (0.30 to 0.87)	<.001	0.71 (−0.37 to 1.79)	.20	0.58 (0.28 to 0.88)	<.001	−0.35 (−0.97 to 0.27)	.27
Autoimmune or cholestatic	−0.03 (−0.14 to 0.07)	.55	−0.15 (−0.62 to 0.33)	.54	0.03 (−0.09 to 0.14)	.66	−0.20 (−0.44 to 0.04)	.10
Baseline decompensation	1.80 (1.49 to 2.12)	<.001	2.16 (0.90 to 3.42)	<.001	1.75 (1.36 to 2.14)	<.001	1.81 (1.22 to 2.40)	<.001
Ascites	1.84 (1.56 to 2.12)	<.001	1.94 (0.81 to 3.07)	<.001	1.91 (1.56 to 2.25)	<.001	1.67 (1.15 to 2.19)	<.001
Hepatic encephalopathy	0.85 (0.59 to 1.10)	<.001	1.12 (0.08 to 2.16)	.04	0.70 (0.39 to 1.02)	<.001	1.10 (0.63 to 1.57)	<.001
Hepatocellular carcinoma	0.13 (−0.01 to 0.28)	.06	−0.09 (−0.35 to 0.17)	.50	0.16 (−0.01 to 0.32)	.07	0.10 (−0.21 to 0.40)	.55
Charlson comorbidity index								
0	−0.73 (−1.05 to −0.40)	<.001	−0.66 (−1.96 to 0.64)	.32	−0.78 (−1.20 to −0.37)	<.001	−0.24 (−0.79 to 0.32)	.41
1	−0.23 (−0.49 to 0.03)	.09	0.52 (−0.52 to 1.56)	.32	−0.17 (−0.50 to 0.16)	.32	−0.50 (−0.98 to −0.01)	.04
2-3	0.46 (0.19 to 0.73)	<.001	0.78 (−0.12 to 1.68)	.09	0.56 (0.23 to 0.89)	<.001	−0.02 (−0.57 to 0.53)	.95
≥4	0.50 (0.24 to 0.76)	<.001	−0.65 (−1.48 to 0.19)	.13	0.39 (0.08 to 0.70)	.01	0.75 (0.20 to 1.30)	.008
Insurance								
Commercial	0.03 (−0.27 to 0.33)	.84	1.38 (0.15 to 2.61)	.03	0.15 (−0.27 to 0.56)	.49	0.44 (0.13 to 0.76)	.006
Medicare	1.02 (0.66 to 1.37)	<.001	0.28 (−0.65 to 1.21)	.56	0.60 (0.22 to 0.98)	.002	−0.33 (−0.74 to 0.08)	.12
Medicaid	−0.47 (−0.77 to −0.18)	.002	−0.90 (−2.22 to 0.43)	.19	−0.24 (−0.64 to 0.17)	.25	0.01 (−0.19 to 0.22)	.90
Other	−0.58 (−0.82 to −0.33)	<.001	−0.76 (−1.91 to 0.39)	.20	−0.51 (−0.85 to −0.17)	.003	−0.13 (−0.33 to 0.07)	.20

### Outcomes

#### Mortality

During follow-up, 3026 patients (32.7%) died: 126 (21.5%) of those younger than 40 years, 1924 (31.9%) of those aged 40 to 64 years, and 976 (36.8%) of those aged 65 years and older. Corresponding mortality rates were 6.4 (95% CI, 5.4-7.6), 9.9 (95% CI, 9.5-10.4), and 16.2 (95% CI, 15.2-17.2) per 100 person-years, respectively (*P* < .001) (eTable 2 in the Supplement). The higher mortality rate in those aged 65 years and older was attenuated after multivariable adjustment (hazard ratio [HR], 1.10; 95% CI, 0.98-1.23). These differences in mortality associated with age were present in patients with both compensated and decompensated cirrhosis, although the differences were more apparent in patients with compensated cirrhosis (eFigure 1 in the Supplement). Corresponding 1-, 2-, and 5-year cumulative survival rates are shown in eTable 3 in the Supplement. Mortality rates decreased during the study period (11.9 [95% CI, 10.7-13.1] per 100 person-years in 2004 vs 10.0 [95% CI, 8.1-12.2] per 100 person-years in 2014; annual adjusted HR, 0.87; 95% CI, 0.86-0.88) (Table 4). Compared with cirrhosis due to viral hepatitis, alcoholic cirrhosis was associated with reduced mortality (adjusted HR, 0.89; 95% CI, 0.80-0.98), and NASH was associated with increased overall mortality (adjusted HR, 1.44; 95% CI, 1.30-1.60) in both bivariable and multivariable analyses (eTable 4 in the Supplement). The reduced mortality associated with alcohol was more apparent for those with decompensated cirrhosis; the increased mortality associated with NASH was more apparent for those with compensated cirrhosis (eFigure 2 in the Supplement). This reduction in mortality was present across age groups and different cirrhosis causes and persisted after multivariable adjustment.

**Table 4.** Temporal Trends in Clinical Outcomes

Outcome by Cause of Cirrhosis	Incidence Rate (95% CI) per 100 Person-Years			HR or SHR (95% CI)	
	2004-2006	2007-2011	2012-2014	Crude	Adjusted^a^
Mortality	11.7 (11.0-12.4)	11.0 (10.5-11.6)	9.9 (9.0-10.8)	0.90 (0.89-0.91)	0.87 (0.86-0.88)
Alcohol	10.3 (9.2-11.5)	9.2 (8.4-10.1)	8.2 (7.0-9.6)	0.89 (0.87-0.91)	0.86 (0.84-0.88)
Viral	9.4 (8.5-10.4)	9.8 (9.0-10.6)	9.0 (7.7-10.5)	0.93 (0.91-0.95)	0.90 (0.88-0.92)
NASH or other	22.1 (19.7-24.8)	18.2 (16.6-19.9)	15.2 (12.9-17.8)	0.87 (0.85-0.88)	0.84 (0.82-0.86)
Autoimmune or cholestatic	12.3 (8.6-17.7)	8.7 (6.1-12.4)	6.3 (3.1-12.5)	0.84 (0.77-0.91)	0.84 (0.76-0.92)
Transplant	0.3 (0.2-0.5)	0.3 (0.2-0.4)	0.3 (0.2-0.5)	0.90 (0.84-0.97)	0.88 (0.81-0.94)
Alcohol	0.3 (0.1-0.6)	0.4 (0.3-0.7)	0.3 (0.1-0.7)	0.93 (0.84-1.03)	0.92 (0.83-1.03)
Viral	0.4 (0.2-0.6)	0.2 (0.2-0.4)	0.3 (0.2-0.8)	0.89 (0.79-1.01)	0.86 (0.76-0.98)
NASH or other	0.2 (0.0-0.6)	0.2 (0.1-0.4)	0.0 (0.0-0.0)	0.81 (0.65-1.01)	NA^b^
Autoimmune or cholestatic	0.8 (0.2-3.3)	1.4 (0.6-3.5)	0.8 (0.1-5.6)	0.92 (0.76-1.12)	1.04 (0.74-1.46)
Hepatocellular carcinoma	1.6 (1.4-1.9)	1.8 (1.6-2.1)	1.8 (1.4-2.3)	1.01 (0.97-1.04)	1.01 (0.98-1.05)
Alcohol	1.1 (0.7-1.6)	0.9 (0.6-1.2)	1.0 (0.6-1.8)	0.96 (0.88-1.04)	0.96 (0.87-1.05)
Viral	2.3 (1.9-2.9)	2.9 (2.5-3.4)	3.1 (2.3-4.3)	1.03 (0.99-1.08)	1.03 (0.99-1.07)
NASH or other	0.6 (0.3-1.3)	0.8 (0.5-1.3)	0.8 (0.3-1.8)	0.99 (0.88-1.12)	0.97 (0.85-1.10)
Autoimmune or cholestatic	0.5 (0.1-3.3)	2.8 (1.4-5.6)	2.3 (0.6-9.1)	1.11 (0.92-1.33)	1.11 (0.76-1.62)
Decompensation^c^	7.7 (7.0-8.4)	10.8 (10.1-11.5)	15.0 (13.5-16.7)	1.04 (1.02-1.06)	1.04 (1.02-1.06)
Alcohol	7.1 (6.0-8.4)	11.2 (9.9-12.6)	17.3 (14.7-20.4)	1.06 (1.03-1.09)	1.06 (1.02-1.09)
Viral	9.3 (8.2-10.4)	12.2 (11.2-13.4)	15.2 (12.9-17.9)	1.02 (1.00-1.05)	1.02 (1.00-1.05)
NASH or other	4.0 (3.0-5.5)	6.6 (5.4-8.0)	11.7 (9.0-15.1)	1.06 (1.01-1.11)	1.06 (1.01-1.12)
Autoimmune or cholestatic	7.6 (4.5-12.8)	9.5 (6.3-14.3)	10.8 (5.4-21.7)	1.04 (0.93-1.15)	1.05 (0.94-1.18)
Ascites^c^	5.6 (5.0-6.2)	8.3 (7.8-9.0)	11.7 (10.4-13.1)	1.07 (1.05-1.09)	1.07 (1.05-1.09)
Alcohol	4.8 (3.9-5.8)	7.8 (6.8-8.9)	13.2 (11.0-16.0)	1.10 (1.06-1.14)	1.10 (1.06-1.14)
Viral	6.9 (6.1-7.9)	9.9 (9.0-10.9)	12.4 (10.3-14.8)	1.06 (1.03-1.08)	1.06 (1.03-1.08)
NASH or other	3.2 (2.3-4.6)	5.1 (4.1-6.4)	8.0 (5.9-10.9)	1.06 (1.00-1.12)	1.07 (1.00-1.13)
Autoimmune or cholestatic	4.6 (2.4-8.8)	8.0 (5.2-12.4)	9.5 (4.5-19.9)	1.13 (1.01-1.28)	1.12 (0.98-1.27)
Hepatic encephalopathy^c^	3.6 (3.1-4.0)	4.7 (4.2-5.1)	5.4 (4.5-6.4)	1.02 (1.00-1.05)	1.02 (0.99-1.05)
Alcohol	3.3 (2.6-4.2)	5.7 (4.8-6.6)	6.8 (5.3-8.8)	1.06 (1.02-1.10)	1.04 (1.00-1.09)
Viral	4.3 (3.6-5.0)	5.1 (4.5-5.8)	5.3 (4.0-7.0)	1.01 (0.97-1.04)	1.01 (0.97-1.05)
NASH or other	1.6 (1.0-2.6)	1.8 (1.3-2.7)	3.4 (2.1-5.5)	1.01 (0.93-1.09)	0.99 (0.90-1.09)
Autoimmune or cholestatic	4.4 (2.3-8.4)	3.4 (1.8-6.6)	2.6 (0.7-10.5)	0.94 (0.80-1.10)	0.97 (0.82-1.15)
Variceal bleeding^c^	1.6 (1.3-1.9)	1.9 (1.7-2.3)	2.3 (1.8-3.0)	0.99 (0.95-1.02)	1.00 (0.96-1.04)
Alcohol	1.4 (1.0-2.0)	2.7 (2.1-3.4)	2.4 (1.6-3.8)	1.02 (0.96-1.08)	1.00 (0.94-1.07)
Viral	2.1 (1.6-2.6)	2.1 (1.7-2.6)	2.9 (2.0-4.2)	0.96 (0.91-1.01)	0.97 (0.92-1.03)
NASH or other	0.2 (0.0-0.8)	0.5 (0.3-1.0)	1.4 (0.7-2.9)	1.14 (0.96-1.36)	1.20 (0.97-1.47)
Autoimmune or cholestatic	1.5 (0.5-4.7)	0.4 (0.1-2.6)	0.0 (0.0-0.0)	0.77 (0.38-1.56)	NA^b^

Abbreviations: HR, hazard ratio; NA, not applicable; NASH, nonalcoholic steatohepatitis; SHR, subhazard ratio.

^a^ Multivariable models were adjusted for sex, cirrhosis cause, decompensation, Charlson comorbidity index, insurance, and year of cohort entry. For the mortality outcomes, HRs are reported; for other outcomes, SHRs are reported. The HRs and SHRs refer to the relative changes per year.

^b^ Adjusted values could not be calculated because of the small number of outcomes.

^c^ Decompensation outcomes were assessed only in those with compensated cirrhosis at baseline.

#### Transplant

Liver transplant was performed for 88 patients (1.0%), at similar rates across age groups (eTable 2 in the Supplement). Transplant rates were low (0.3 [95% CI, 0.3-0.4] per 100 person-years) and decreased during the study period (annual adjusted subhazard ratio [SHR], 0.88; 95% CI, 0.81-0.94) (Table 4). Transplant rates were lower in patients with NASH (adjusted SHR, 0.32; 95% CI, 0.12-0.82) than in patients with viral hepatitis (eTable 4 in the Supplement).

#### Hepatocellular Carcinoma

The incidence of HCC was low in patients younger than 40 years, with an absolute incidence rate of 0.5 (95% CI, 0.2-0.9) per 100 person-years; 7 (88%) of these patients who developed HCC had viral hepatitis (eTable 2 in the Supplement). Older patients had a similar absolute risk of HCC compared with those aged 40 to 64 years, but after multivariable adjustment, the risk was increased (SHR, 1.58; 95% CI, 1.13-2.22). This increased risk appeared to be associated with the cause of cirrhosis. In those without viral hepatitis, the adjusted SHR for older patients was 1.24 (95% CI, 0.79-1.95); in those with viral hepatitis, the adjusted SHR was 1.82 (95% CI, 1.19-2.78). The rate of HCC in those aged 40 to 64 years with viral hepatitis was 2.7 (95% CI, 2.3-3.0) per 100 person-years, and in those aged 65 years and older it was 4.4 (95% CI, 3.2-5.9) per 100 person-years compared with all patients without viral hepatitis (0.9 [95% CI, 0.8-1.1] per 100 person-years). The rate of incident HCC remained constant (Table 4).

#### Decompensation

Patients younger than 40 years with compensated cirrhosis had a lower rate of subsequent decompensation compared with older patients (adjusted SHR, 0.78; 95% CI, 0.62-0.99), predominantly associated with a lower rate of hepatic encephalopathy (adjusted SHR, 0.53; 95% CI, 0.36-0.78) (eTable 2 in the Supplement). Nonalcoholic steatohepatitis was associated with lower rates of incident ascites (adjusted SHR, 0.46; 95% CI, 0.37-0.56), hepatic encephalopathy (adjusted SHR, 0.38; 95% CI, 0.28-0.50), variceal bleeding (adjusted SHR, 0.28; 95% CI, 0.16-0.47), and overall decompensation (adjusted SHR, 0.51; 95% CI, 0.43-0.60) (eTable 4 in the Supplement). Rates of decompensation increased over the study period, largely because of an increase in the incidence of ascites (Table 4). Rates of hepatic encephalopathy and variceal bleeding did not change over time.

### Sensitivity Analyses

Restricting the definition of NASH led to an increase in NASH prevalence (0.38% per year; 95% CI, 0.21%-0.55%; *P* < .001) but also to an increased prevalence among those aged 65 years and older (0.48% per year; 95% CI, 0.05%-0.91%; *P* = .03) as opposed to the nonsignificant decrease shown in Table 3. The excess mortality associated with NASH in the primary analysis (eTable 4 in the Supplement) was largely associated with patients without a recorded cause of cirrhosis; those with the restricted NASH definition had unadjusted mortality rates similar to those for the other causes (10.5 [95% CI, 9.1-12.1] per 100 person-years) and reduced mortality after multivariable adjustment (HR, 0.82; 95% CI, 0.69-0.97). Other outcomes were unaffected by the alternative NASH definition.

## Discussion

In this statewide cohort study of patients with newly diagnosed cirrhosis over a decade, we found increases in the proportion of those younger than 40 years and 65 years and older, as well as increases in the proportions of patients with alcoholic cirrhosis and NASH, with a decrease in the proportion of patients with viral hepatitis. The increasing burden of alcoholic liver disease in younger adults is consistent with the findings of several other studies. One study of US death certificates^4^ showed an increase in cirrhosis-related mortality in young adults from 2009 to 2016 driven by alcoholic liver disease. Another study^25^ of commercially insured adults in the United States showed an increase in the prevalence of alcoholic cirrhosis, with this increase most pronounced in younger patients. Hospitalization costs for cirrhosis have also increased in recent years because of an increasing burden of alcoholic cirrhosis.^26^ More broadly, in a nationally representative sample, high-risk drinking and alcohol use disorders increased from 2001 to 2013 across all age groups, with the highest absolute risk among younger adults.^8^ Future projections based on epidemiologic data also support an increasing effect of alcohol on the population with cirrhosis.^27^ Our study provides data showing that these increases in alcohol use, cirrhosis prevalence, mortality, and costs are also reflected in an increasing number of new diagnoses of alcoholic cirrhosis in younger adults. The long-term costs of this shift include lost productivity in those of prime working age, as well as costs associated with liver disease surveillance (eg, HCC and varices) in younger patients with longer life expectancies.^28,29^ These data support the allocation of resources to reduce the burden of alcohol use disorder, which is increasingly leading to advanced liver disease.

In addition to the increasing numbers of younger patients with alcoholic cirrhosis, we also found increased numbers of older patients and those with NASH-related cirrhosis. These findings reflect the obesity epidemic and are consistent with studies showing an increasing prevalence of NASH and increasing numbers of older patients and those with NASH-related cirrhosis pursuing liver transplant.^5,9,10,30^ Importantly, these shifts have implications for the future because patients with new diagnoses now may require transplant years later. Other studies have projected such increases in NASH- and obesity-related transplants decades after increases in obesity prevalence.^10^ These trends may also negatively affect the population of patients who need transplant because their increasing comorbidity burden will render them less suitable for transplant.^31^ Notably, we found an increasing comorbidity burden in this study, particularly in older patients.

In contrast to these increases, there was a relative decrease in the number of patients aged 40 to 64 years with newly diagnosed cirrhosis and in the number of cases of cirrhosis due to viral hepatitis. These trends likely reflect the aging of the cohort of patients with hepatitis C (most of whom were born 1945-1965), whose ages ranged from 39 in 2004 to 69 in 2014. It is important to note that the absolute numbers of those aged 40 to 64 years and those with viral hepatitis did increase until 2011; however, these increases were smaller than those in the comparator groups, resulting in a relative decrease. However, from 2011 onward, the absolute numbers of these patients decreased by 13%. These decreases cannot be attributed to highly effective treatment regimens for hepatitis C, which were not available until 2013; rather, these figures likely reflect the fact that the incidence of new hepatitis C infections has been decreasing since the 1980s.^32^ With the current widespread treatment of hepatitis C, we would anticipate more drastic reductions of viral hepatitis–related cirrhosis and its complications in the future.^11^

A significant finding was the decreasing mortality rate, which has been seen in other studies.^33,34^ One study^35^ posited a shift of inpatient mortality to the immediate postdischarge setting, but nevertheless found reduced 1-year mortality. Although this study does not allow us to definitively comment on reasons for this improvement, it may be associated with improved care. For hospitalized patients with cirrhosis, timely endoscopy and paracentesis have been linked to decreasing mortality.^33,36^ Others have found increasing use of antibiotics, an intervention with proven mortality benefit, for patients with cirrhosis and upper gastrointestinal tract bleeding.^37^ The mortality improvement in our study occurred despite an increasing prevalence of decompensation both at diagnosis and during follow-up (mostly ascites). The reason for this increase in baseline decompensation is unclear, but it may reflect the shift in causes of cirrhosis away from viral hepatitis and toward alcohol and NASH. Notably, baseline decompensation was much more common among patients with alcoholic and NASH cirrhosis compared with patients with viral hepatitis. Patients with viral hepatitis may be more likely to undergo liver fibrosis assessment at an asymptomatic phase, whereas in patients with alcoholism and NASH, liver disease may be diagnosed only after symptoms of end-stage liver disease occur. The decrease in mortality also occurred despite extremely low rates of transplant, which also decreased during the study period. This low transplant rate highlights the importance of focusing on quality care to improve outcomes, because transplant alone is unlikely to achieve these goals on a population scale. Future work should continue to explore reasons for these mortality trends.

The prevalence of baseline HCC and incident HCC during follow-up remained stable over time. This finding is in contrast to those of other studies^38,39^ showing an increase in HCC incidence, driven primarily by hepatitis C. However, these studies analyzed patients treated in the Veterans Affairs system, who have a greater prevalence of viral hepatitis; therefore, those results may not be generalizable to the general population. In contrast, our study includes patients from multiple health systems and payers, and our findings may be more generalizable. One study^2^ of Surveillance, Epidemiology, and End Results Program data did show decreasing HCC incidence and mortality rates in particular racial and ethnic groups in recent years. Another notable finding is the extremely low prevalence (1%) and incidence (0.5 per 100 person-years) of HCC in younger patients, which has been seen elsewhere.^40^ If confirmed, these data could support less stringent HCC surveillance in the growing population of younger patients with cirrhosis.

### Limitations

There are several limitations to this study. Although the Indiana Network for Patient Care covers multiple health systems, it is not a true population-based cohort and does not have complete coverage of Indiana. However, it is the nation’s oldest, largest, and most comprehensive regional health information exchange, and its inclusion of multiple payers including Medicare and Medicaid makes it a unique data source to follow a large, diverse population longitudinally. This is in contrast to other studies that are limited to single payers or health systems.^25,38^ In addition, although Indiana has relatively high rates of alcohol use disorders and alcoholic cirrhosis–associated mortality, the state is not an outlier compared with other states, which further enhances the generalizability of the findings. We used diagnostic and procedural codes to identify cirrhosis and other factors that can be associated with misclassification errors. However, when possible, we used codes that had been previously validated. Furthermore, limiting the cohort to only incident cirrhosis diagnoses (with a 1-year run-in period) does not represent the entire population with cirrhosis. Indeed, the observed low transplant rate likely reflects that most patients with cirrhosis receive a diagnosis shortly after entering the health care system. However, specifying the cohort in this way allows for the identification of incident diagnoses more accurately than including all cirrhosis diagnoses, and it provides a more robust way to assess outcomes. Notably, the mortality estimates in this study are similar to those of prior natural history studies of cirrhosis.^12^ Other strengths of the study include the large sample size, broad geographic representation throughout Indiana, and linkage to the Social Security death index, which supports accurate mortality estimates.

## Conclusions

This study found a shifting profile of the population of those with newly diagnosed cirrhosis that is likely to affect clinical care and future outcomes. These data can help inform contemporary natural history projections for different causes of cirrhosis in different age groups. Of particular interest are the increased number of diagnoses in younger and older patients and increases in the numbers of cases of alcoholic cirrhosis and NASH. We also found changes in outcomes, notably a decreasing mortality rate. Together, these data support the allocation of resources toward the prevention of alcoholic liver disease and the treatment of NASH, as well as understanding ways to better care for these increasing populations. In addition, existing models of care that have focused on populations of predominantly viral hepatitis–induced cirrhosis have been useful; however, new models to better address the unique needs of younger and older patients with cirrhosis (eg, dedicated care focusing on treatment of alcohol use disorders and comorbidities associated with NASH) are needed to continue to improve outcomes.

## Notes

Supplement. **eTable 1. **Claims-Based Definitions **eTable 2. **Incidence of Clinical Outcomes Based on Age **eTable 3.** Cumulative Survival According to Baseline Decompensation Status, Age, and Cause of Cirrhosis **eTable 4.** Incidence of Clinical Outcomes Based on Cause of Cirrhosis **eFigure 1.** Mortality According to Age **eFigure 2.** Mortality According to Cause of Cirrhosis
