# Error in Byline

**DOI:** 10.1001/jamanetworkopen.2019.13673

**Published:** 2019-10-02

In the Original Investigation titled “Trends in Characteristics, Mortality, and Other Outcomes of Patients With Newly Diagnosed Cirrhosis,”^1^ published June 28, 2019, in the byline, the middle initial was missing for Archita P. Desai, MD. This article has been corrected.^1^

## References

[1] Orman ES, Roberts A, Ghabril M, Trends in characteristics, mortality, and other outcomes of patients with newly diagnosed cirrhosis. JAMA Netw Open. 2019;2(6):. doi:10.1001/jamanetworkopen.2019.6412 31251379 PMC6604080

