# Supplementary material for: Trends in Characteristics, Mortality, and Other Outcomes of Patients With Newly Diagnosed Cirrhosis
Source: JAMA Netw Open. 2019 Jun 28;2(6):e196412. doi: 10.1001/jamanetworkopen.2019.6412 (PMC6604080; doi:10.1001/jamanetworkopen.2019.6412)
Supplement: Supplement. — eTable 1. Claims-Based Definitions eTable 2. Incidence of Clinical Outcomes Based on Age eTable 3. Cumulative Survival According to Baseline Decompensation Status, Age, and Cause of Cirrhosis eTable 4. Incidence of Clinical Outcomes Based on Cause of Cirrhosis eFigure 1. Mortality According to Age eFigure 2. Mortality According to Cause of Cirrhosis [file jamanetwopen-2-e196412-s001.pdf]

Supplementary Online Content

Orman ES, Roberts A, Ghabril M, et al. Trends in characteristics, mortality, and other outcomes of patients with newly diagnosed cirrhosis. *JAMA Netw Open*. 2019;2(6):e196412. doi:10.1001/jamanetworkopen.2019.6412

eTable 1. Claims-Based Definitions
eTable 2. Incidence of Clinical Outcomes Based on Age
eTable 3. Cumulative Survival According to Baseline Decompensation Status, Age, and Cause of Cirrhosis
eTable 4. Incidence of Clinical Outcomes Based on Cause of Cirrhosis
eFigure 1. Mortality According to Age
eFigure 2. Mortality According to Cause of Cirrhosis

This supplementary material has been provided by the authors to give readers additional information about their work.

© 2019 Orman ES et al. *JAMA Network Open*

eTable 1: Claims-Based Definitions

	ICD-9-CM	CPT	Laboratory
Alcoholic liver disease	571.0; 571.1; 571.2; 571.3; 291.x; 303.x; 305.0x		
Any solid organ transplant	00.91; 00.92; 00.93; 33.5; 33.50; 33.51; 33.52; 33.6; 37.5; 37.51; 46.97; 50.5; 50.51; 50.59; 52.8; 52.80; 52.81; 52.82; 52.83; 55.6; 55.61; 55.69; 996.8; V42; V58.44; E878.0	32851; 32852; 32853; 32854; 33935; 33945; 44135; 44136; 47135; 47136; 48554; 50360; 50365	
Ascites	789.59		
Autoimmune hepatitis	571.42		
Cirrhosis	456.0; 456.1; 456.2; 456.21; 567.23; 571.2; 571.5; 572.2; 572.3; 572.4		
Dyslipidemia	272.0; 272.1; 272.2; 272.4; 272.5; 272.9		
Hepatic encephalopathy	572.2		
Hepatitis B	070.2; 070.20; 070.21; 070.22; 070.23; 070.30; 070.31; 070.32; 070.33		HBsAg
Hepatitis C	070.41; 070.44; 070.51; 070.54; 070.7; 070.70; 070.71		Anti-HCV<br>HCV RNA
Hepatocellular carcinoma	155.0		
Hypertension	401; 401.0; 401.1; 401.9; 402.0; 402.1; 402.9; 403.0; 403.1; 403.9; 404; 404.0; 404.1; 404.9; 278.0; 278.00; 278.01; 278.02; 278.1; V77.8; 783.1; 278.02		
Impaired fasting glucose/diabetes mellitus	250; 790.2; 790.21; 790.22; 790.29		
Liver transplant	50.5; 50.51; 50.59	47135; 47136	
Nonalcoholic steatohepatitis	571.8		
Obesity	278.0; 278.00; 278.01; 278.1; V77		

© 2019 Orman ES et al. *JAMA Network Open*

Paracentesis	54.91	49080; 49081	
Primary biliary cholangitis	571.6		
Primary sclerosing cholangitis	576.1		
Variceal hemorrhage	456.0; 456.20		

Abbreviations: ICD-9-CM, International Classification of Diseases, Clinical Modification; CPT, Current Procedural Terminology; HBsAg, hepatitis B surface antigen; anti-HCV, hepatitis C antibody; HCV RNA, hepatitis C ribonucleic acid.

© 2019 Orman ES et al. *JAMA Network Open*

eTable 2. Incidence of Clinical Outcomes Based on Age

	Person-years	Events	Incidence rate[a] (95% CI)	Crude HR/SHR (95% CI)	Adjusted HR/SHR[b] (95% CI)
**Mortality**					
**<40**	1,975	126	6.4 (5.4-7.6)	0.66 (0.55-0.79)	0.66 (0.55-0.80)
**40-64**	19,435	1,924	9.9 (9.5-10.4)	Ref	Ref
**≥65**	6,037	976	16.2 (15.2-17.2)	1.43 (1.33-1.55)	1.10 (0.98-1.23)
**Transplant**					
**<40**	1,975	6	0.3 (0.1-0.7)	1.06 (0.46-2.45)	1.21 (0.52-2.82)
**40-64**	19,435	61	0.3 (0.2-0.4)	Ref	Ref
**≥65**	6,037	21	0.3 (0.2-0.5)	0.88 (0.54-1.44)	1.38 (0.67-2.83)
**Hepatocellular carcinoma**					
**<40**	1,690	8	0.5 (0.2-0.9)	0.27 (0.13-0.54)	0.36 (0.18-0.72)
**40-64**	16,218	304	1.9 (1.7-2.1)	Ref	Ref
**≥65**	4,691	84	1.8 (1.4-2.2)	0.84 (0.66-1.07)	1.58 (1.13-2.22)
**Decompensation[c]**					
**<40**	1,129	82	7.3 (5.9-9.0)	0.75 (0.59-0.93)	0.78 (0.62-0.99)
**40-64**	11,597	1,188	10.2 (9.7-10.8)	Ref	Ref
**≥65**	3,603	402	11.2 (10.1-12.3)	0.92 (0.83-1.03)	1.08 (0.93-1.26)
**Ascites[c]**					
**<40**	1,180	68	5.8 (4.5-7.3)	0.79 (0.62-1.01)	0.86 (0.67-1.10)
**40-64**	12,245	950	7.8 (7.3-8.3)	Ref	Ref
**≥65**	3,745	318	8.5 (7.6-9.5)	0.93 (0.82-1.06)	1.11 (0.93-1.33)
**Hepatic encephalopathy[c]**					
**<40**	1,272	29	2.3 (1.6-3.3)	0.55 (0.38-0.79)	0.53 (0.36-0.78)
**40-64**	12,922	577	4.5 (4.1-4.8)	Ref	Ref
**≥65**	3,887	180	4.6 (4.0-5.4)	0.88 (0.75-1.04)	1.19 (0.95-1.50)
**Variceal bleeding[c]**					
**<40**	1,262	19	1.5 (1.0-2.4)	0.81 (0.51-1.29)	0.82 (0.50-1.33)
**40-64**	13,131	267	2.0 (1.8-2.3)	Ref	Ref
**≥65**	3,990	56	1.4 (1.1-1.8)	0.58 (0.44-0.78)	0.94 (0.64-1.37)

[a]Per 100 person-years.

© 2019 Orman ES et al. *JAMA Network Open*

[b]Multivariable models were adjusted for sex, cause of cirrhosis, decompensation, Charlson comorbidity index, insurance, and year of cohort entry. For the mortality outcomes, hazard ratios (HR) are reported; for other outcomes, subhazard ratios (SHR) are reported.
[c]Decompensation outcomes were assessed in only those with compensated cirrhosis at baseline.
Abbreviations: HR, hazard ratio; SHR, subhazard ratio; CI, confidence interval.

© 2019 Orman ES et al. *JAMA Network Open*

eTable 3: Cumulative Survival According to Baseline Decompensation Status, Age, and Cause of Cirrhosis

	1-year survival (95% CI)	2-year survival (95% CI)	5-year survival (95% CI)
**Compensated**			
**<40**	94.5 (91.5-96.5)	92.8 (89.4-95.1)	85.4 (80.1-89.4)
**40-64**	90.6 (89.6-91.4)	84.7 (83.4-85.8)	72.1 (70.4-73.8)
**≥65**	84.1 (82.2-85.8)	75.9 (73.6-78.0)	57.6 (54.5-60.6)
**Decompensated**			
**<40**	73.1 (66.3-78.8)	64.5 (57.0-71.1)	55.6 (47.2-63.2)
**40-64**	65.1 (62.9-67.3)	57.1 (54.7-59.5)	41.7 (38.8-44.6)
**≥65**	62.6 (59.1-66.0)	52.4 (48.4-56.2)	34.1 (28.7-39.5)
**Compensated**			
**Alcohol**	91.4 (90.0-92.6)	86.5 (84.7-88.0)	73.9 (71.3-76.3)
**Viral**	91.5 (90.3-92.5)	85.2 (83.7-86.5)	70.8 (68.7-72.9)
**NASH/other**	80.3 (78.0-82.3)	71.7 (69.0-74.2)	58.1 (54.7-61.3)
**Autoimmune/cholestatic**	92.8 (87.7-95.9)	88.2 (82.1-92.3)	75.1 (65.7-82.3)
**Decompensated**			
**Alcohol**	73.1 (70.4-75.6)	64.9 (61.8-67.7)	51.1 (47.2-54.8)
**Viral**	65.5 (62.3-68.6)	55.5 (52.0-58.9)	36.8 (32.8-40.9)
**NASH/other**	52.7 (48.9-56.3)	46.2 (42.2-50.1)	33.4 (28.6-38.3)
**Autoimmune/cholestatic**	59.7 (45.2-71.6)	38.7 (23.6-53.7)	27.5 (12.1-45.5)

Abbreviations: CI, confidence interval; NASH, nonalcoholic steatohepatitis.

eTable 4: Incidence of Clinical Outcomes Based on Cause of Cirrhosis

	Person-years	Events	Incidence rate[a] (95% CI)	Crude HR/SHR (95% CI)	Adjusted HR/SHR[b] (95% CI)
**Mortality**					
**Alcohol**	9,444	881	9.3 (8.7-10.0)	0.94 (0.86-1.02)	0.89 (0.80-0.98)
**Viral**	12,565	1,198	9.5 (9.0-10.1)	Ref	Ref
**NASH/other**	4,722	879	18.6 (17.4-19.9)	1.69 (1.55-1.85)	1.44 (1.30-1.60)
**Autoimmune/cholestatic**	716	68	9.5 (7.5-12.1)	0.94 (0.74-1.20)	1.01 (0.76-1.33)
**Transplant**					
**Alcohol**	9,444	35	0.4 (0.3-0.5)	1.13 (0.72-1.79)	1.10 (0.66-1.82)
**Viral**	12,565	39	0.3 (0.2-0.4)	Ref	Ref
**NASH/other**	4,722	6	0.1 (0.1-0.3)	0.29 (0.13-0.69)	0.32 (0.12-0.82)
**Autoimmune/cholestatic**	716	8	1.1 (0.6-2.2)	3.39 (1.58-7.26)	3.87 (1.72-8.70)
**Hepatocellular carcinoma**					
**Alcohol**	7,881	76	1.0 (0.8-1.2)	0.35 (0.27-0.45)	0.30 (0.23-0.41)
**Viral**	10,336	281	2.7 (2.4-3.1)	Ref	Ref
**NASH/other**	3,792	28	0.7 (0.5-1.1)	0.25 (0.17-0.36)	0.22 (0.15-0.35)
**Autoimmune/cholestatic**	590	11	1.9 (1.0-3.4)	0.67 (0.37-1.24)	0.59 (0.29-1.19)
**Decompensation[c]**					
**Alcohol**	5,083	541	10.6 (9.8-11.6)	0.92 (0.83-1.03)	0.97 (0.86-1.09)
**Viral**	7,776	891	11.5 (10.7-12.2)	Ref	Ref
**NASH/other**	2,970	195	6.6 (5.7-7.6)	0.50 (0.43-0.59)	0.51 (0.43-0.60)
**Autoimmune/cholestatic**	501	45	9.0 (6.7-12.0)	0.77 (0.57-1.03)	0.82 (0.59-1.13)
**Ascites[c]**					
**Alcohol**	5,406	406	7.5 (6.8-8.3)	0.83 (0.73-0.93)	0.86 (0.75-0.98)
**Viral**	8,236	744	9.0 (8.4-9.7)	Ref	Ref
**NASH/other**	3,010	150	5.0 (4.2-5.8)	0.48 (0.40-0.57)	0.46 (0.37-0.56)
**Autoimmune/cholestatic**	519	36	6.9 (5.0-9.6)	0.75 (0.54-1.04)	0.83 (0.58-1.19)
**Hepatic encephalopathy[c]**					
**Alcohol**	5,662	281	5.0 (4.4-5.6)	1.04 (0.90-1.21)	1.12 (0.95-1.31)
**Viral**	8,790	423	4.8 (4.4-5.3)	Ref	Ref
**NASH/other**	3,085	62	2.0 (1.6-2.6)	0.36 (0.28-0.48)	0.38 (0.28-0.50)

© 2019 Orman ES et al. *JAMA Network Open*

**Autoimmune/cholestatic**	545	20	3.7 (2.4-5.7)	0.76 (0.49-1.18)	0.80 (0.49-1.30)
**Variceal bleeding[c]**					
**Alcohol**	5,766	126	2.2 (1.8-2.6)	1.00 (0.80-1.25)	1.17 (0.92-1.50)
**Viral**	8,956	195	2.2 (1.9-2.5)	Ref	Ref
**NASH/other**	3,111	17	0.5 (0.3-0.9)	0.22 (0.13-0.36)	0.28 (0.16-0.47)
**Autoimmune/cholestatic**	550	4	0.7 (0.3-1.9)	0.33 (0.12-0.87)	0.24 (0.06-0.97)

[a]Per 100 person-years.
[b]Multivariable models were adjusted for sex, cause of cirrhosis, decompensation, Charlson comorbidity index, insurance, and year of cohort entry. For the mortality outcomes, hazard ratios (HR) are reported; for other outcomes, subhazard ratios (SHR) are reported.
[c]Decompensation outcomes were assessed in only those with compensated cirrhosis at baseline.
Abbreviations: NASH, nonalcoholic steatohepatitis; HR, hazard ratio; SHR, subhazard ratio; CI, confidence interval.

© 2019 Orman ES et al. *JAMA Network Open*

eFigure 1. Mortality According to Age

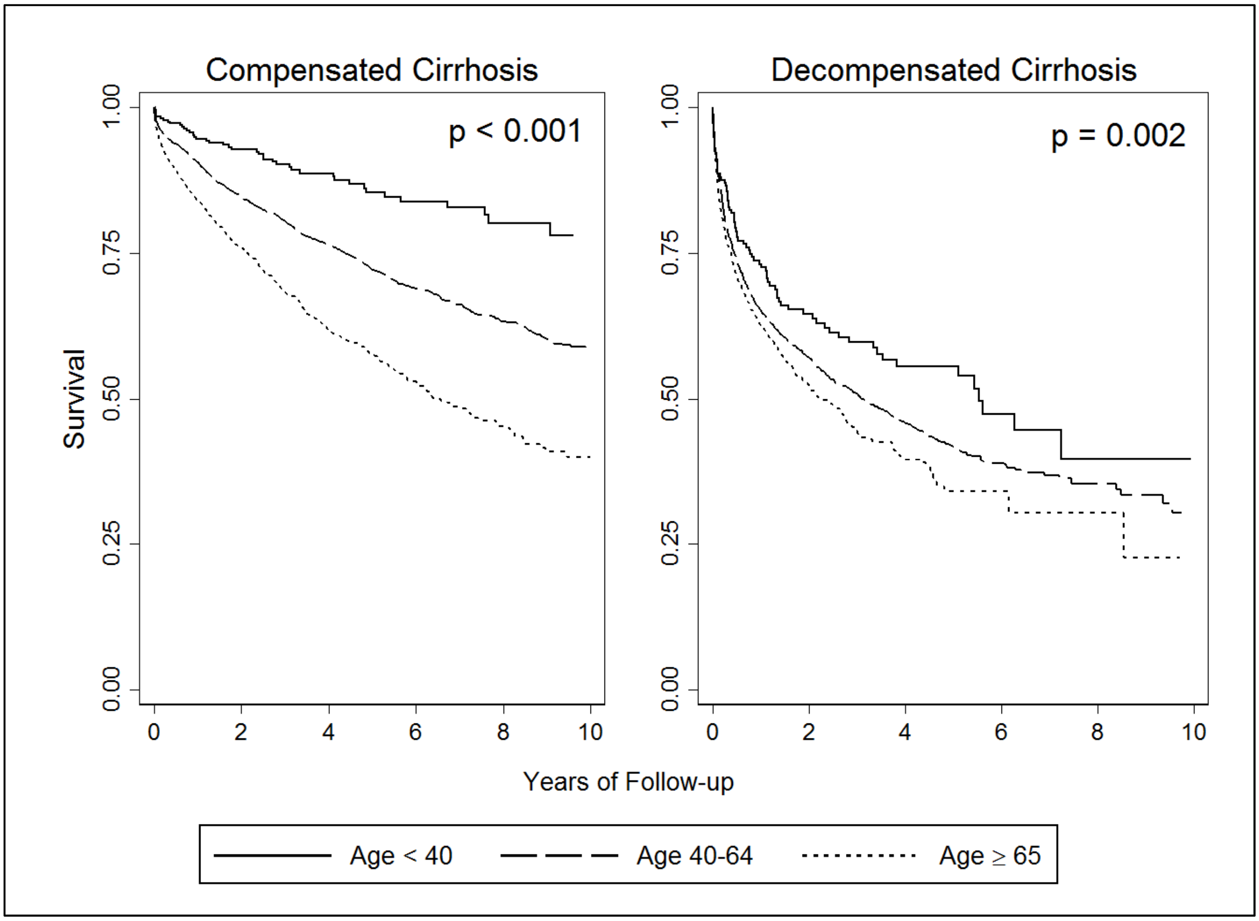

© 2019 Orman ES et al. *JAMA Network Open*

eFigure 2. Mortality According to Cause of Cirrhosis

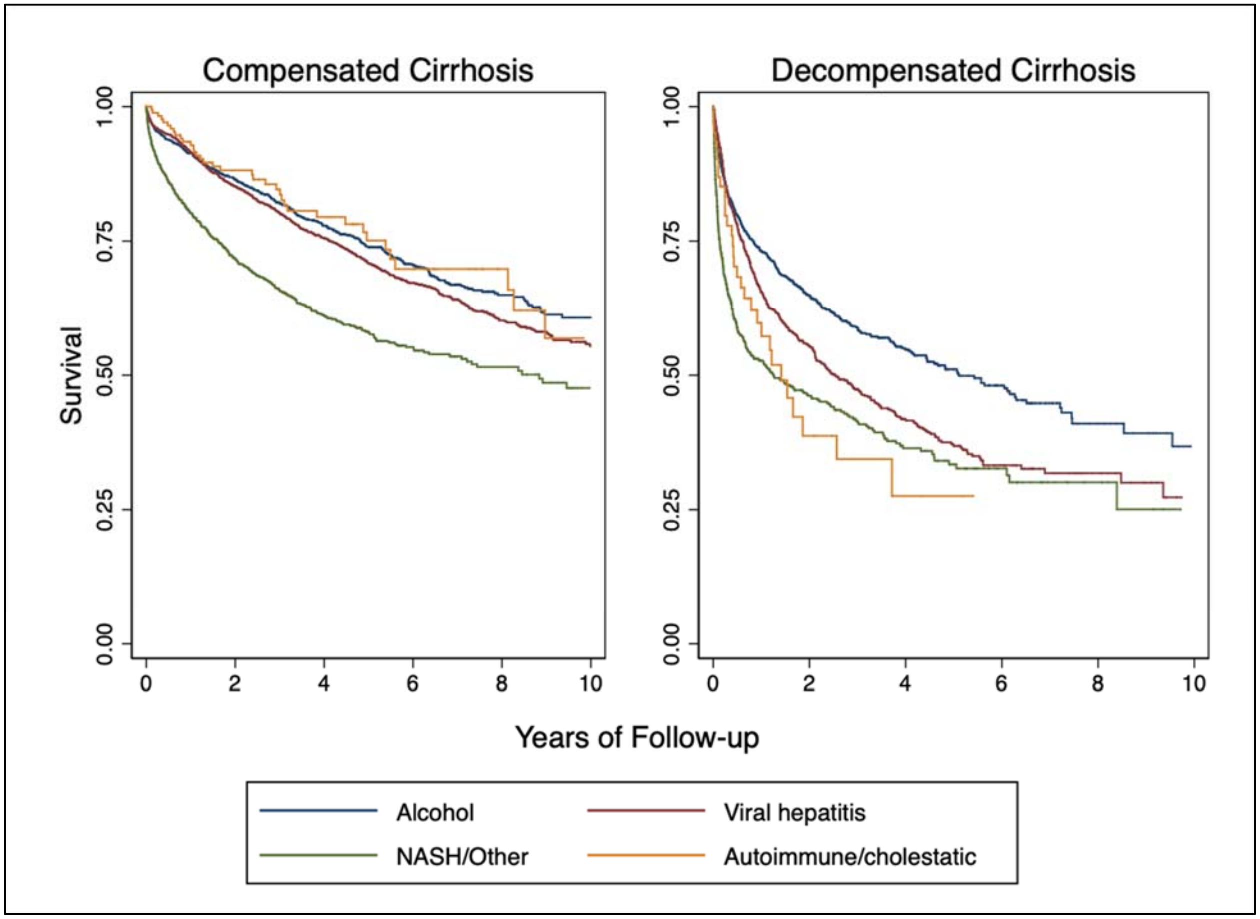

© 2019 Orman ES et al. *JAMA Network Open*
